# Comparative sequence analysis reveals an intricate network among *REST*, *CREB *and miRNA in mediating neuronal gene expression

**DOI:** 10.1186/gb-2006-7-9-r85

**Published:** 2006-09-26

**Authors:** Jie Wu, Xiaohui Xie

**Affiliations:** 1 Department of Biomedical Engineering, Boston University, Boston, Massachusetts 02215, USA; 2 Broad Institute of MIT and Harvard, 7 Cambridge Center, Cambridge, Massachusetts 02142, USA

## Abstract

**Background:**

Two distinct classes of regulators have been implicated in regulating neuronal gene expression and mediating neuronal identity: transcription factors such as *REST/NRSF *(RE1 silencing transcription factor) and *CREB *(cAMP response element-binding protein), and microRNAs (miRNAs). How these two classes of regulators act together to mediate neuronal gene expression is unclear.

**Results:**

Using comparative sequence analysis, here we report the identification of 895 sites (NRSE) as the putative targets of *REST*. A set of the identified NRSE sites is present in the vicinity of the miRNA genes that are specifically expressed in brain-related tissues, suggesting the transcriptional regulation of these miRNAs by *REST*. We have further identified target genes of these miRNAs, and discovered that *REST *and its cofactor complex are targets of multiple brain-related miRNAs including miR-124a, miR-9 and miR-132. Given the role of both *REST *and miRNA as repressors, these findings point to a double-negative feedback loop between *REST *and the miRNAs in stabilizing and maintaining neuronal gene expression. Additionally, we find that the brain-related miRNA genes are highly enriched with evolutionarily conserved cAMP response elements (CRE) in their regulatory regions, implicating the role of *CREB *in the positive regulation of these miRNAs.

**Conclusion:**

The expression of neuronal genes and neuronal identity are controlled by multiple factors, including transcriptional regulation through *REST *and post-transcriptional modification by several brain-related miRNAs. We demonstrate that these different levels of regulation are coordinated through extensive feedbacks, and propose a network among *REST*, *CREB *proteins and the brain-related miRNAs as a robust program for mediating neuronal gene expression.

## Abstract 2

Using comparative sequence analysis, a network among REST, CREB and brain-related miRNAs is propsed to mediate neuronal gene expression.

## Background

Regulation of gene expression is critical for nervous system development and function. The nervous system relies on a complex network of signaling molecules and regulators to orchestrate a robust gene expression program that leads to the orderly acquisition and maintenance of neuronal identity. Identifying these regulators and their target genes is essential for understanding the regulation of neuronal genes and elucidating the role of these regulators in neural development and function.

The transcriptional repressor *REST *(RE1 silencing transcription factor, also called neuron-restrictive silencer factor or *NRSF*) plays a fundamental role in regulating neuronal gene expression and promoting neuronal fate [1,2]. *REST *contains a zinc-finger DNA-binding domain and two repressor domains interacting with corepressors *CoREST *and *mSin3a*. The corepressors additionally recruit the methyl DNA-binding protein *MeCP2*, histone deacetylases (*HDAC*), and other silencing machinery, which alter the conformation of chromatin resulting in a compact and inactive state [3-6]. *REST *is known to target many neuronal genes, and is pivotal in restricting their expression exclusively in neuronal tissues by repressing their expression in cells outside the nervous system. Recent work also points to *REST *as a key regulator in the transition from embryonic stem cells to neural progenitors and from neural progenitors to neurons [7]. The role of *REST *in nervous system development is intriguingly manifested by its expression, which is lower in neural stem/progenitor cells than in pluripotent stem cells, and becomes minimal in post-mitotic neurons [7]. The expression of *REST *is shown to be regulated by retinoic acid; however, other forms of regulatory mechanisms are unknown.

Another important class of regulators implicated in neuronal gene expression control and neuronal fate determination is the microRNA (miRNA) [8-10]. MiRNAs are an abundant class of endogenous approximately 22-nucleotide RNAs that repress gene expression post-transcriptionally. Hundreds of miRNAs have been identified in almost all metazoans including worm, fly, and mammals, and are believed to regulate thousands of genes by virtue of base pairing to 3' untranslated regions (3'UTRs) of the messages. Many of the characterized miRNAs are involved in developmental regulation, including the timing and neuronal asymmetry in worm; growth control and apoptosis in fly; brain morphogenesis in zebrafish; and hematopoetic and adipocyte differentiation, cardiomyocyte development, and dendritic spine development in mammals [8,11,12]. Based on data from a recent survey [13], we note that the human genome contains about 326 miRNA genes, many of which are highly or specifically expressed in neural tissues [14]. The function of the brain-related miRNAs and the mechanisms underlying their transcriptional control are beginning to emerge [12,15-17].

In addition to *REST *and miRNAs, many other classes of regulators might also be involved in controlling neuronal gene expression. This control could be carried out through a variety of mechanisms, such as changing chromatin state, affecting mRNA stability and transport, and post-translational modifications. Here we focus specifically on regulation through *REST *and miRNAs.

To gain a better understanding of how *REST *and miRNAs regulate neuronal gene expression, we took the initial step of producing a reliable list of genes targeted by *REST *and several brain-related miRNAs using computational approaches. A list of these target genes should be informative in unraveling the function of these regulators. Moreover, we anticipate that a global picture of the target genes may provide a clue as to how *REST *and miRNAs act together to coordinate neuronal gene expression programs and promote neuronal identity.

*REST *represses target genes by binding to an approximately 21-nucleotide binding site known as NRSE (neuron-restrictive silencer element, also called RE1), which is present in the regulatory regions of target genes. Previously, several genome-wide analyses of NRSE sites have been carried out [6,18,19]. These analyses used pattern-matching algorithms to search for sequences matching a consensus derived from known *REST *binding sites. The most recent work identified 1,892 sites in the human genome [19]. However, there are several factors limiting the utilities of the pattern-matching algorithms. Most notably, transcriptional factors can bind with variable affinities to sequences that are allowed to vary at certain positions. Consequently, methods based on consensus sequence matching are likely to miss target sites with weaker binding affinities. Indeed, it has been noted that both *L1CAM *and *SNAP25 *genes contain an experimentally validated NRSE site that diverges from the NRSE consensus [19], and was not identified in the previous analyses. In addition, even sequences perfectly matching the NRSE consensus could occur purely by chance, and therefore do not necessarily imply that they are functional. Given the vast size of the human genome, random matches could significantly add to the false positive rate of a prediction. For example, in the most recent analysis, it was estimated that 41% of the 1,892 predicted sites occur purely by chance, and likely represent false positives [19].

We have developed a method to systematically identify candidate NRSE sites in the human genome without these two main limitations of the previous methods. To address the first limitation, we utilized a profile-based approach, which computes the overall binding affinity of a site to *REST *without requiring strict matching of each base to the NRSE consensus. To reduce false positives, we rely on comparative sequence analysis to identify only sites that are conserved in orthologous human, mouse, rat and dog regions [20-23].

MiRNAs repress gene expression by base-pairing to the messages of protein-coding genes for translational repression or message degradation. The pairing of miRNA seeds (nucleotides 2 to 7 of the miRNAs) to messages is necessary and appears sufficient for miRNA regulation [24-26]. This enables the prediction of miRNA targets by searching for evolutionarily conserved 7-nucleotide matches to miRNA seeds in the 3'UTRs of the protein-coding genes [21,27-30]. We have generated a list of predicted target genes for several brain-related miRNAs by searching for seed-matches perfectly conserved in mammalian 3'UTRs.

Additionally, we have sought to understand the mechanisms controlling the expression of brain-related miRNAs. To this end, we have used comparative analysis to identify sequence motifs that are enriched and conserved in the regulatory regions of these miRNAs across several mammals.

## Results

### Identification of 895 NRSE sites in human with a false positive rate of 3.4%

**Figure 1:**
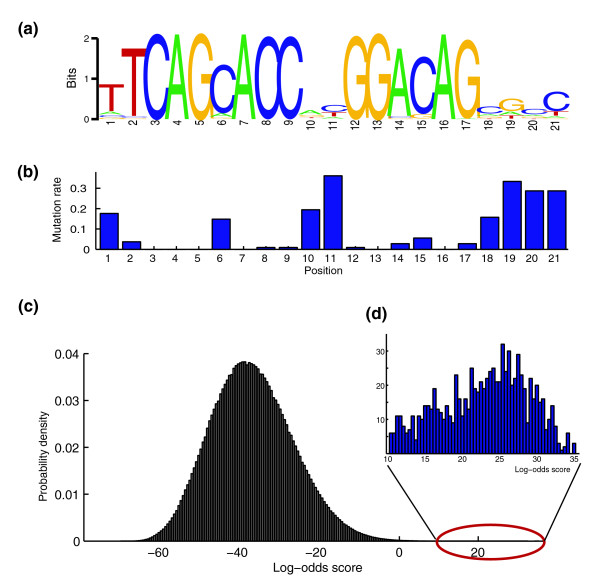
NRSE profile and distribution of log-odds score. **(a) **Position weight matrix of NRSE at 21 positions constructed from 38 known NRSE sites. The y-axis represents the information content at each position. **(b) **The average number of bases mutated in orthologous regions of mouse, rat or dog at each position of the NRSE profile, when the nonhuman sequences are compared with the corresponding human site. The number is calculated based on the 37 known NRSE sites that can be aligned in the four species. **(c) **Distribution of background NRSE log-odds score calculated over regulatory regions (from upstream 5 kb to downstream 5 kb around each transcriptional start) of all human protein-coding genes. **(d) **Distribution of NRSE log-odds score on 895 identified NRSE sites.

First, we curated from the literature a list of experimentally validated NRSE sites in the human genome [18,19], including 38 sites with site lengths of 21 nucleotides (see supplementary table 1 in Additional data file 1). Based on the 38 known sites, we derived a profile (also called a position weight matrix) on the distribution of different nucleotides at each position of NRSE. The profile shows an uneven contribution to the binding of the *REST *protein from each of the 21 positions (Figure 1a). The positions 2 to 9 and 12 to 17 nucleotides, which will be referred as 'core positions' of NRSE, are much less variable than the remaining positions.

Next we examined the conservation properties of the known NRSE sites. To carry this out, we extracted orthologous regions of these sites in three other fully sequenced mammalian genomes (mouse, rat and dog) [31-34], and generated an alignment for each site in the four species (see supplementary table 1 in Additional data file 1). The alignment data show that the NRSE sites are highly conserved across the mammalian lineages: out of the 38 reference sites, only one cannot be detected in other mammals. We further examined the conservation of NRSE by counting the number of bases mutated in other species from the aligned human site at each of its 21 positions. Similar to the profile, conservation levels at different NRSE positions are highly non-uniform (Figure 1b). However, the conservation levels at different positions are remarkably well correlated with the NRSE profile: highly constrained positions show much stronger conservation in orthologous species than those with higher variability. The core positions are highly constrained and permit few mutations. Among the 37 aligned sites, all core positions contain fewer than two mutations and no insertions or deletions in any of the other species when compared with a human site. By contrast, in a random control, only 0.47 out of the 38 sites are expected to be called conserved with the same criteria. Therefore, the functional NRSE sites demonstrate a 78-fold increase of evolutionary conservation, suggesting the usefulness of evolutionary conservation as an efficient tool for detecting NRSE sites.

We then used the profile to search the entire human genome for sites that are better described by the profile than other background models. For each candidate 21-nucleotide window in the genome, we calculated a log-odds score quantifying how well the site fits to the NRSE profile (see Materials and methods). The overall distribution of the log-odds scores computed over the regulatory regions of all protein-coding genes in humans is shown in Figure 1c, which follows a normal distribution (mean = -37; standard deviation (SD) = 10). We were interested in sites with scores significantly higher than the bulk of the overall distribution: over the entire human genome, we identified 171,152 sites with log-odds scores above 5 (corresponding to 4.2 SDs away from the mean).

The next step was to examine orthologous sequences of these sites in other mammals and filter the list to 1,498 sites based on two criteria: (a) the log-odds scores at the orthologous sites of mouse, rat and dog are also greater than 5, and (b) the number of bases mutated from the corresponding human sequence at the core positions is fewer than two in any of the orthologous sites. The criterion (b) is based on the conservation properties of the known NRSE sites described above.

We then estimated the number of sites that could be discovered purely by chance. For this purpose, we generated a cohort of control profiles with the same base composition and the same information contents as those of the NRSE profile, and searched the instances of the control profiles using the same procedure. Only 328 sites were found for the control profiles, suggesting that approximately 78% of the 1,498 sites are likely to be *bona fide *NRSE sites. To balance the need for an even smaller rate of false positives, we further identified 895 sites with log-odds scores above 10 in all aligned species. Only 30 sites are expected by chance, suggesting a false positive rate of 3.4%. The distribution on the log-odds scores of these sites falls distinctly to the far right of the bulk of the background distribution (Figure 1c). These sites are distributed across all chromosomes of the human genome and include 37 out of the 38 known NRSE sites that we have curated.

Next we identified the nearest protein-coding genes located around each of the 895 candidate NRSE sites. Over 60% of these genes have NRSE sites within 20 kb of their transcriptional starts (Supplementary figure 1 in Additional data file 1), while a few NRSE sites are located more than 150 kb away from genes, suggesting the possibility of long-range interactions. To study the properties of these genes further, we generated a list of 566 genes that contain at least one NRSE site within 100 kb of their transcriptional start sites (see supplementary website [35]). Interestingly, 75 (13.2%) of the genes contain more than one NRSE site in their regulatory regions. For instance, *NSF *(N-ethylmaleimide-sensitive factor) contains as many as four NRSE sites in its regulatory region in a segment of sequence of less than 100 base pairs; another gene *NPAS4 *(neuronal PAS domain protein 4) contains three NRSE sites spread over a region of 3 kb.

**Figure 2:**
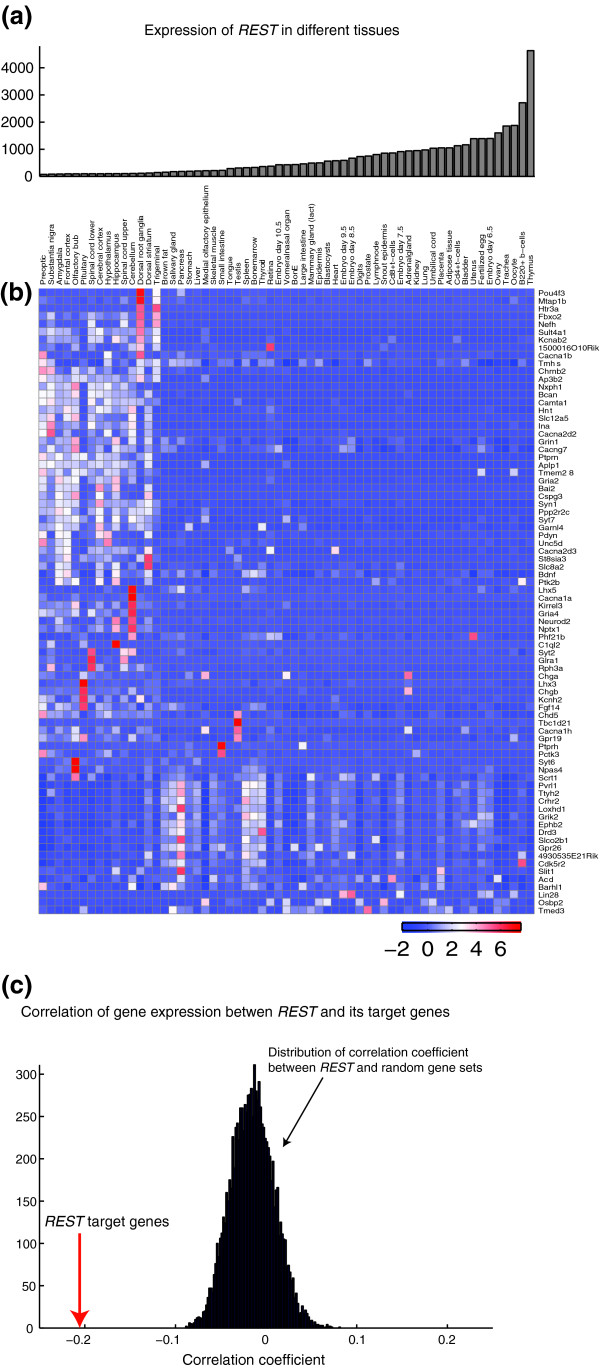
Gene expression patterns of predicted *REST *targets in 61 mouse tissues. **(a) **Expression of gene *REST *in different tissues. **(b) **Expression of predicted *REST *targets. Only 80 genes with top NRSE log-odds scores are shown. The tissues in (a) are arranged in the same order as those in (b). The genes shown in (b) are clustered based on hierarchical clustering such that genes sharing similar expression patterns are grouped together. **(c) **Mean correlation coefficient between *REST *and each of the genes shown in (b). Also shown is the distribution of these values when the genes in (b) are randomly chosen.

If the predicted genes are *bona fide REST *targets, we would expect that the expression of these genes should inversely correlate with the expression of *REST*. To test this, we examined the expression of these genes and *REST *across a battery of mouse tissues in a dataset generated previously [36]. The tissue gene expression dataset contains 409 of the predicted target genes. It confirms that *REST *is expressed at low levels in brain-related tissues, and at much higher levels in non-neuronal tissues (Figure 2a). In contrast to the expression profile of *REST*, most of the predicted *REST *target genes are specifically expressed in brain-related tissues (Figure 2b). We calculated the correlation coefficient between *REST *and each of the predicted target genes: the mean correlation coefficient for the genes shown in Figure 2b is -0.21, which is much lower (P value = 2.2e^-16^) than what is expected by chance (Figure 2c). Using a stringent threshold (See Materials and methods), we screened out 188 (46% of all 409 genes, 5.4-fold enrichment) genes that demonstrate specific expression in brain-related tissues. A list of these genes and their expression profiles across different tissues is shown in Additional data file 1, supplementary figure 2.

**Figure 3:**
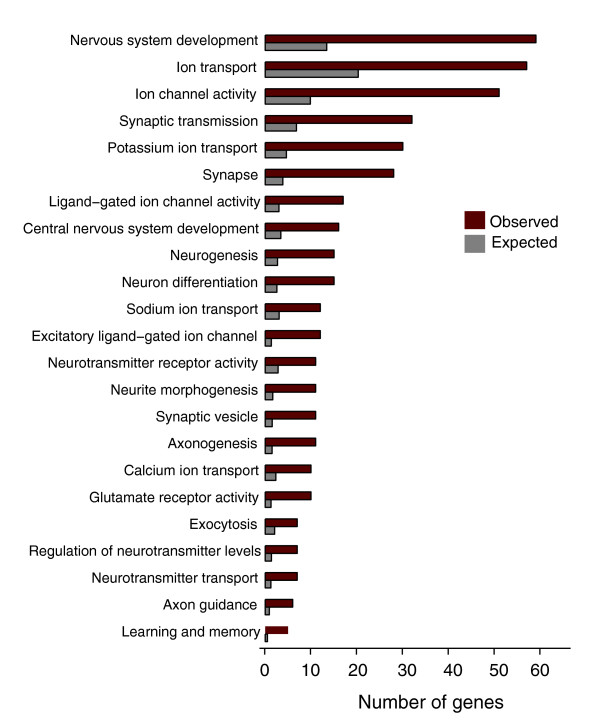
Enriched functional categories for predicted *REST *target genes. Each row represents one function category, and shows the observed number of *REST *target genes contained in that category and the number of genes expected purely by chance.

We then examined the functional annotation of all 566 predicted *REST *target genes. Specifically we were aiming to test if these target genes are enriched in any of the functional categories specified in gene ontology. Based on an annotation provided in [37], we found that the gene set is highly enriched with genes implicated in nervous system development and function (Figure 3). For example, 51 genes (5.2-fold enrichment, P value = 1.3e^-22^) encode ion channel activity, and 28 genes (7.3-fold enrichment, P value = 6.6e^-17^) are involved in synaptic functions. Interestingly, the list also contains a large number of genes (60, 4.4-fold enrichment and P value = 2.1e^-22^) implicated in nervous system development; 15 genes are involved in neuronal differentiation, which include a set of important transcription factors such as *NeuroD1*, *NeuroD2*, *NeuroD4*, *LMX1A*, *SOX2 *and *DLX6*.

However, we also observed some genes that do not seem to encode obvious neural-specific functions. This is consistent with what we observed when examining gene expression patterns for these genes (Figure 2b): a significant portion of them show specific expression in non-neuronal tissues such as brown fat, pancreas, spleen and thyroid (Figure 2b). Interestingly, in most of the tissues the expression of *REST *is also low (Figure 2a), consistent with the role of *REST *as a transcriptional repressor. The extent to which *REST *contributes to the function of other cell types is unclear. A recent study identified *REST *as a tumor suppressor gene in epithelia cells [38]. Together with our findings, this may suggest that *REST *could potentially regulate a set of genes not necessarily specific to neuronal functions. Alternatively, the observed expression of some *REST *target genes in non-neuronal tissues might be due to other confounding factors, such as the heterogeneous cell population in these tissues, added levels of regulation caused by transcriptional regulators which themselves are targeted by *REST*, and the potential regulation by miRNAs, which we will discuss in more detail later.

Thus, using a profile constructed from 38 known NRSE sites and requiring evolutionary conservation in other mammalian species, we have identified 895 sites in the human genome with an estimated false positive rate of 3.4%. We have identified protein-coding genes near these elements, and found that most of these genes are expressed specifically in neuronal tissues.

### Brain-related miRNAs in the vicinity of the NRSE sites

We noticed that there is a set of miRNAs that are located in close proximity to the predicted 895 NRSE sites in the human genome (Table 1). This includes 10 miRNA genes that are located within 25 kb of at least one NRSE site, where no protein-coding genes can be found nearby. Three of the miRNAs, miR-124a, miR-9 and miR-132, have further experimental support for targeting by *REST*, as demonstrated in a chromatin immunoprecipitation analysis by Conaco *et al*. [39]. Additionally, we discovered that miR-29a, miR-29b and miR-135b are also located in the vicinity of the NRSE sites. All these 10 miRNA genes are located in intergenic regions, and are transcribed with their own promoters. We also found that there is a set of miRNA genes likely regulated by *REST *indirectly through the promoters of protein-coding genes that host these miRNAs. These miRNA genes are located in the introns of protein-coding genes, which themselves are predicted *REST *targets. It is known that miRNAs located inside protein-coding genes are often cotranscribed with the host, and spliced out only after transcription. The set of miRNAs include miR-153 within *PTPRN*, miR-346 within glutamate receptor *GRID1*, and miR-218 within *SLIT3*.

Overall, we identified 16 miRNA genes that are potentially regulated by *REST *(Table 1) directly or indirectly through their protein-coding hosts. Interestingly, most of these miRNAs are expressed in the brain, and some of them show brain-specific/enriched expression patterns. In a recent survey of several miRNA expression-profiling studies, Cao *et al*. generated a list of 34 miRNAs that demonstrate brain-specific/enriched expression in at least one study [14]. The 16 miRNA genes we identified correspond to 13 unique miRNA mature products. Out of the 13 miRNAs, eight (62%) are contained in the list of 34 brain-specific/enriched miRNAs summarized by Cao *et al*., which is about sixfold enrichment when compared with what is expected by chance (34 out of 319 all miRNAs, 10.6%). Among the six miRNAs not included in the list of 34 brain-related miRNAs, mir-29 has been demonstrated to show dynamic expression patterns during brain development, and is strongly expressed in glial cells during neural cell specification [14,40]; mir-346, mir-95 and mir-455 are contained in the introns of (and share the same strand as) their protein-coding hosts, which themselves are specifically expressed in brain-related tissues (supplementary figure 5 in Additional data file 1). It is unclear how these miRNAs and their host genes appear to demonstrate different expression patterns.

In summary, this suggests that similar to neuronal genes, a set of brain-related miRNAs are likely under the control of *REST *as well. *REST *might play an important role in repressing the expression of these miRNAs in cells outside the nervous system.

### Identification of target genes for each of the brain-related miRNAs

MiRNAs have been suggested to regulate the expression of thousands of genes. Our next step was to seek to identify genes that are targeted by the set of brain-related miRNAs mentioned above. We used an approach similar to previous analyses [21,27], and identified candidate targets by searching for conserved matches of the miRNA seeds (2 to 7 nucleotides of the miRNA) in the 3'UTRs of the protein-coding genes. To reduce the rate of false positives, we required the seed to be conserved not only in eutherian mammals as used in the previous analysis, but also in marsupials. For this purpose, we first generated an aligned 3'UTR database in the orthologous regions of the human, mouse, rat, dog and opossum genomes (HMRDO). Then we searched the aligned 3'UTRs for conserved 7-nucleotide sequences that could form a perfect Watson-Crick pairing to each of the miRNA seeds. This effort lead to hundreds of predicted targets for the brain-related miRNAs, including 315 targets for miR-124a, 273 targets for miR-9, and 80 targets for miR-132. The complete list of predicted target genes for each of the brain-related miRNAs can be viewed at the supplementary website [35].

We examined the expression of the predicted target genes in different mouse tissues. The expression profile of the predicted target genes for each of the miRNAs across different tissues is shown in the supplementary website [35]. Interestingly, we noticed that the brain-related miRNAs target many genes that are highly transcribed in neural tissues (supplementary figure 3 in Additional data file 1). For instance, among 191 genes targeted by mir-124a that have been profiled across different tissues, 45 (23.6%) are specifically expressed in brain-related tissues, which is 2.8-fold enrichment of that which would be expected by chance (8.54%). The enrichment also holds true for mir-9 in that 25.8% of its target genes show brain-specific expression (threefold enrichment). The coexistence of the predicted target genes and the miRNAs in the same tissues suggests that the brain-related miRNAs are likely involved in extensive regulation of a large number of neuronal genes.

### Evidence for a double-negative feedback loop between *REST *complex and brain-related miRNAs

**Figure 4:**
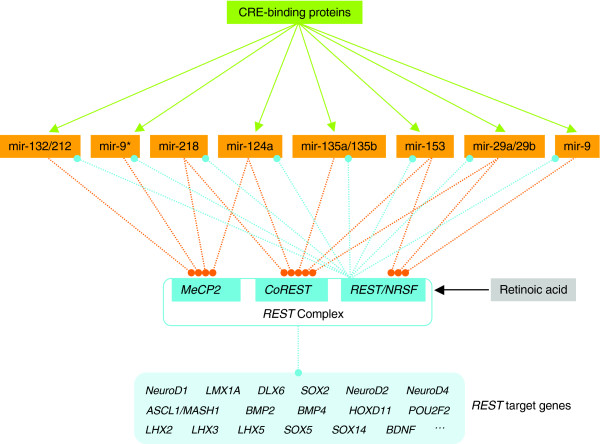
Schematic diagram of the interactions among *REST*, *CREB *and miRNAs. The three classes of regulators are represented by different colors, with the *REST *complex shown in blue, miRNAs shown in orange, and *CREB *family proteins shown in green. A list of *REST *target genes is shown in light blue. Positive interactions are indicated with solid lines with arrows, while negative interactions are denoted with dotted lines with filled circles.

Interestingly, the miRNA target list includes several proteins forming the core *REST *complex, such as *MeCP2 *and *CoREST*. For example, *MeCP2 *is targeted by numerous brain-specific miRNAs including miR-132, miR-212, miR-9*, miR-218, and miR-124a. Similarly, corepressor *CoREST *is targeted by miR-124a, miR-218, miR-135b, and miR-153 (Figure 4).

As to the *REST *itself, our initial analysis did not identify any miRNA that could bind to its 3'UTR. However, a closer examination indicates that gene *REST *harbors a much longer 3'UTR transcript, not annotated by any gene prediction programs (Additional data file 1, supplementary figure 4). This longer 3'UTR is supported by three pieces of evidence: 1) multiple ESTs detected in this region; 2) high levels of conservation across all mammalian species, and even chicken; and 3) a perfectly conserved poly-adenylation site (AATAAA) in all mammals at the end of the new transcript.

Based on the new 3'UTR transcript, we performed the target prediction again and discovered that *REST *itself is also targeted by several brain-related miRNAs including miR-9, miR-29a, and miR-153. Together with the discovery of regulation by *REST *on these miRNAs, this suggests the existence of an extensive double feedback loops between the *REST *complex and the brain-related miRNAs.

We notice that the 3'UTR of the *REST *also harbors predicted target sites for several miRNAs that do not seem to have obvious neuronal-specific functions. Out of the seven unique target sites (conserved in HMRDO), three sites are not contained in the list of 34 brain-specific/enriched miRNAs curated by Cao *et al*. [14], including one site targeted by mir-93 family, one site targeted by mir-25 family, and one site targeted by mir-377. Both mir-93 and mir-25 are enriched in non-neuronal tissues such as spleen and thymus [41]. This seems to reinforce the observation of expression patterns for the predicted protein-coding targets of *REST*, where we also noticed a set of target genes specifically expressed in non-neuronal tissues (Figure 2). We speculate that *REST *might be involved in the regulation of genes outside the nervous systems.

### cAMP response element binding protein (*CREB*) is a potential positive regulator of the brain-related miRNAs

Next we sought to understand the regulatory machinery controlling the expression of the set of brain-related miRNAs. Besides the negative regulation by *REST*, we are particularly interested in factors that positively regulate the expression of these miRNAs. Given the scarcity of data on the regulation of miRNA in general, we decided to take an unbiased approach to look for short sequence motifs enriched in the regulatory regions of these miRNAs.

Since few primary transcripts of the miRNA genes are available, we decided to examine a relatively big region (from upstream 10 kb to downstream 5 kb) around each of the miRNAs. On the other hand, however, using big regions significantly increases the difficulty of detecting any enriched motifs. We therefore resorted to comparative sequence analysis again, by searching only for sequence motifs present in aligned regions of the four mammals. For this purpose, we generated a list of all 7-nucleotide motifs, and for each motif we counted the number of conserved and total instances in those regions, and computed a score quantifying the enrichment of the conserved instances (see Materials and methods section. The analysis yielded 35 motifs that are significantly enriched in these regions with a P value less than 10^-6 ^(Table 2). The top motif is GACGTCA, which is a consensus cAMP response element (CRE) recognized by *CREB*, a basic leucine zipper transcription factor. We repeated the motif discovery using 6-mer and 8-mer motifs, and consistently identified the CRE element as the most significant motif. For the ten miRNA genes (Table 1) predicted to be directly regulated by *REST*, we found nine containing a conserved CRE site nearby. This set of miRNAs includes miR-124a, miR-9, miR-29a/29b, and miR-132 (Table 3, Figure 4). Although this association is purely computational, a recent study demonstrated experimentally that one of these miRNAs, miR-132, is regulated by *CREB *and is involved in regulating neuronal morphogenesis [42].

**Figure 5:**
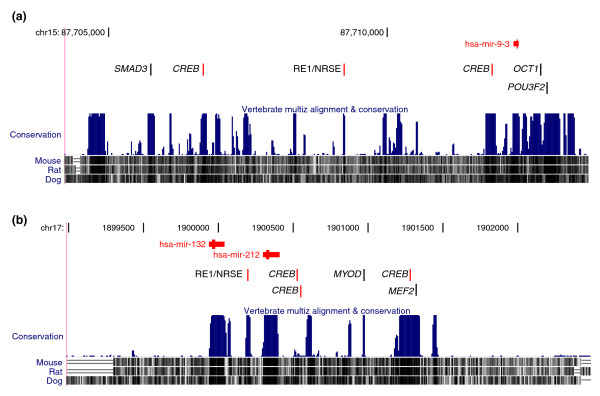
Predicted regulatory elements in the regulatory regions of miRNA genes. The annotation in the regulatory regions of **(a) **miR-9 and **(b) **miR-132/212, are shown. Each panel shows the positions of regulatory elements on a background annotation of genes and sequence conservations extracted from the UCSC genome browser. Not one protein-coding gene is present in both regions. The bottom part of each panel shows the conservation of human sequence when compared with other mammalian species. Aligned human sequences are denoted with vertical lines at aligned positions for mouse, rat and dog, respectively. The track denoted by 'conservation' plots the overall conservation levels of the human sequence in each region. The regulatory elements demonstrate higher levels of conservation and stand out from the background sequences.

In addition to *CREB*, we also identified several other potential regulators such as *E47*, *SMAD3*, *POU3F2*, and *MYOD*. For instance, besides *REST *and *CREB*, miR-9-3 is predicted to be regulated by *SMAD3*, *OCT1*, and *POU3F2 *(Figure 5a), and miR-132 is predicted to be regulated by *MYOD *and *MEF2 *(Figure 5b). Interestingly, a recent study shows that *MEF2 *and *MYOD *control the expression of another miRNA, miR-1, and play an important role in regulating cardiomyocyte differentiation [11]. As well as being expressed in muscle tissues, *MEF2 *is also highly expressed in brain, where it plays an important role in controlling postsynaptic differentiation and in suppressing excitatory synapse number [43]. It would be interesting to examine whether miRNAs are involved in such processes via the regulation by *MEF2*.

Thus, we have identified several transcription factors that potentially regulate the expression of the brain-related miRNAs with *CREB *being the top candidate. It is likely that the expression of the brain-related miRNAs is under rigorous control of these regulators during different developmental stages and in different cell types.

## Discussion

Comparative sequence analysis is a powerful and general tool for detecting functional elements, because these elements are often under strong selective pressure to be preserved, and therefore stand out from neutrally evolving sequences by displaying a greater degree of conservation across related species. In this work, we have relied on comparative genomics to study the regulation of neuronal gene expression, and have identified functional elements for three distinct classes of regulators including *REST*, *CREB*, and miRNAs.

We identified 895 NRSE sites conserved in human, mouse, rat and dog with an estimated false positive rate of 3.4%. The number is significantly lower than 41%, which is the estimated false positive rate in the previous analysis by Bruce *et al*. [19], where across-species conservation criteria were not considered. Moreover, we used a profile-based approach, and were able to identify sites deviating from the NRSE consensus. For instance, we successfully identified two experimentally validated sites in *L1CAM *and *SNAP25 *that deviate from the NRSE consensus and were missed in previous analyses.

A set of the predicted sites is located in close proximity to a set of brain-related miRNA genes. This suggests that similar to the regulation of neuronal genes, many brain-specific miRNAs are likely to be repressed by *REST *in non-neuronal tissues. To help better understand the function of these miRNAs, we have generated a list of predicted target genes for each of the miRNAs. The predicted targets include many genes that are specifically expressed in neural tissues, suggesting the potentially extensive regulation by the miRNAs on these genes.

We discovered that the *REST *corepressor complex itself is targeted by multiple brain-related miRNAs (Figure 4). Together with the repressive role of *REST *on these miRNAs, the analysis points to the existence of a double-negative feedback loop between the transcription factor *REST *and brain-related miRNAs in mediating neuronal gene expression. The double-negative feedback loop is used widely in engineering as a robust mechanism for maintaining the stability of a dynamic system. A two-component system with mutual inhibitions often results in a bistable system in which only one component is active at the resting state, and the active component can be stabilized against noisy perturbations by negative feedbacks. We speculate that the nervous system may utilize this mechanism in restricting the expression of neuronal genes exclusively in neuronal tissues. It has been reported that *REST *is actively transcribed in neural progenitors during neurogenesis [7]. Moreover, there are also reports showing that mRNA of *REST *is present in mature hippocampal neurons, and the mRNA level can be elevated following epileptic insults [44]. If these transcripts are all translated into *REST *proteins, a large number of neuronal genes will be repressed, most likely undesirably. However, little *REST *protein can be detected in neural progenitors, so to what extent the *REST *protein is expressed in the mature hippocampus neurons is unclear. Previously, the proteasomal-dependent pathway was suggested to be involved in the post-translational degradation of the *REST *protein [7]. We suggest that the set of miRNAs targeting *REST *might be an additional mechanism ensuring the removal of *REST *products in neuronal tissues.

We have used gene expression data measured across different tissues to examine the expression patterns of *REST*, its target genes and the brain-related miRNAs. However, there are several confounding factors that might limit the utility of such expression data. First, the tissues typically contain heterogeneous cell types. For instance, the brain tissues are always a mixture of neurons and glials. If a gene is expressed differentially in different cell types, its expression measured at tissue level may become hard to interpret. Second, the expression data may be further confounded by many secondary effects. For example, transcriptional regulators controlled by *REST *may themselves lead to expression changes for a large number of genes. Indeed, many of the predicted *REST *targets are transcription factors, such as *NeuroD1*, *NeuroD2 *and *NeuroD4*, involved in neural differentiation, and several LIM homeobox proteins such as *LHX2*, *LHX3 *and *LHX5*. The measured expression levels are likely a combined effect of several levels of regulation. Third, because of the added levels of regulation by miRNAs, RNA measurement of a gene may not reflect its true expression levels. As we mentioned above, it has been observed that *REST *is transcribed in neural progenitor cells, but little *REST *protein can be detected. Examining protein expression data is certainly more desirable. However, at present we have few high-quality large-scale protein expression data available. Such data might gradually become available in the future with the recent development in protein-microarray technology and progress in proteomic surveys by mass spectrometry.

In additional to *REST*, which is a regulator repressing the set of brain-related miRNAs, we are also interested in identifying the factors positively regulating those miRNAs. We have undertaken an unbiased approach of searching conserved and enriched short motifs in regulatory regions of these miRNAs, and have identified *CREB *as the top candidate regulator. *CREB *is an important transcription factor regulating a wide-range of neuronal functions including neuronal survival, neuronal proliferation and differentiation, process growth, and synaptic plasticity [45,46]. *CREB *can be activated via phosphorylation by multiple extracellular stimuli such as neurotrophins, cytokines, and calcium, as well as a variety of cellular stresses. The discovery of regulation of multiple miRNAs by *CREB *indicates that these miRNAs are potentially expressed in an activity-dependent manner. It would be interesting to examine whether these miRNAs play a role in regulating synapse development and plasticity.

## Conclusion

We have identified 895 putative NRSE sites conserved in human, mouse, rat and dog genomes. A subset of these NRSE sites is present in the vicinity of several brain-related miRNAs, suggesting the transcriptional repression of these miRNAs by *REST*. We have also found that the brain-related miRNAs are enriched with CRE elements in their promoter regions, implicating the role of *CREB *in the positive regulation of these miRNAs. Altogether, the comparative sequences analysis points to an intricate network of transcription activators and repressors acting together with miRNAs in coordinating neuronal gene expression and promoting neuronal identity.

## Materials and methods

### Multiple sequence alignment among human, mouse, rat and dog

We used the whole-genome mammalian alignments generated by the UCSC genome browser [47]. From the whole-genome alignment, we then extracted regions of interest. For instance, we generated the aligned NRSE sequences based on genome coordinates of NRSE sites in human. Similarly, we constructed the aligned 3'UTR database using the coordinates of 3'UTRs of all protein-coding genes. For 3'UTRs, we used five-way alignments (human, mouse, rat, dog and opossum). The annotation of genes and their 3'UTRs are from the collection of known genes deposited in the UCSC genome browser.

### Constructing the NRSE profile and calculation of log-odds score

The NRSE profile was constructed from 38 known NRSE sites each with a site length of 21 nucleotides. We used the 38 sites to compute the frequency of different nucleotides at each position, and generated a position weight matrix representation *P *of the profile, where *p*_*ij *_represents the probability of nucleotide *j *at position *i*. The information content of a profile is defined as *IC*_*i *_= *2*+Σ_*j *_*p*_ij_*log_2_(*p*_*ij*_) for position *i*. For any candidate 21-nucleotide sequence, we then calculated a log-odds score to evaluate how well the sequence matched to the NRSE profile. The log-odds score is defined as *LO *= Σ_*i *_*log*_2_(*p*_*i*, *j*(*i*)_/*b*_*j*(*i*)_) where *j*(*i*) is the nucleotide at position *i *of the sequence, and *b*_*j *_represents the probability of observing nucleotide *j *in a background model. The log-odds score computes the log ratio of two likelihoods, one that the site is generated by the NRSE profile, and the other that the site is generated by a neutral background model. In the neutral background model, we assume each nucleotide is generated independently according to a given nucleotide composition. We estimated the nucleotide composition based on sequences extracted from regulatory regions (5 kb upstream) of all known genes for each of the species separately.

### Analysis of gene expression across different tissues

We used the microarray gene expression data published previously by Su *et al*. [36], which profiled expression patterns of genes across 61 mouse tissues. We postprocessed the dataset and removed any probe with a mean expression level across different tissues of less than 100, and an SD less than 50. For genes containing multiple probes in the array, we used values averaged over different probes to represent the expression level for that gene. In total, 13,743 genes were used for further analysis. For each of the genes, we then normalized their expression values across different tissues such that the mean expression across different tissues was zero and the SD was 1. Based on the normalized values, we then screened out genes with expression values higher than 0.35 in at least one of the brain-related tissues. A total number of 1,174 genes was identified, and we refer to the gene set as the brain-related genes.

### Identification of regulatory motifs for brain-related miRNAs

First we generated a multiple sequence alignment between human, mouse, rat and dog for the region from 10 kb upstream to 5 kb downstream for each miRNA. We then searched the occurrence of all 7-mers in the aligned regions. For each 7-mer, we counted the number of total instances (*N*) in human, and the number of instances (*K*) perfectly conserved in the aligned regions of mouse, rat and dog. We then calculated a Z-score defined as (*K-Np*_0_)/[*Np*_0_(1-*p*_0_)]^1/2^, where *p*_0 _is the background conservation rate. The Z-score measures the number of standard deviations on the number of conserved instances away from what is expected by chance by assuming a binomial model on whether a site is conserved. The Z-score quantifies the enrichment of conserved motifs in the aligned regions. To achieve a significant Z-score, a 7-mer must be highly conserved and occur in high frequencies.

## Additional data files

Supporting figures and tables are available with the online version of this article in Additional data file 1. The identified NRSE sites, the miRNA target genes and other materials mentioned in the article can be viewed at a supplementary website [35].

## Supplementary Material

Additional data file 1 A PDF containing supporting figures and tables. Click here for file

## Figures and Tables

**Table 1:** A list of miRNAs near predicted NRSE elements in the human genome

miRNA	NRSE sequence	Coordinate (hg17)	Distance (bp)	Host gene
mir-124a-1	TTCAGTACCGAAGACAGCGCCC	chr8:9820071-9820092	-21721	-
mir-124a-2	ATCAAGACCATGGACAGCGAAC	chr8:65450519-65450540	-3795	-
mir-124a-3	TTCAACACCATGGACAGCGGAT	chr20:61277903-61277924	-2437	-
mir-9-1	TCCAGCACCACGGACAGCTCCC	chr1:153197524-153197545	5749	-
mir-9-3	CTCAGCACCATGGCCAGGGCCC	chr15:87709202-87709223	-3094	-
mir-132	ATCAGCACCGCGGACAGCGGCG	chr17:1900204-1900225	-202	-
mir-212	ATCAGCACCGCGGACAGCGGCG	chr17:1900204-1900225	165	-
mir-29a	TTCAGCACCATGGTCAGAGCCA	chr7:130007654-130007675	11117	-
mir-29b-1	TTCAGCACCATGGTCAGAGCCA	chr7:130007654-130007675	11838	-
mir-135b	TTCAGCACCTAGGACAGGGCCC	chr1:202159913-202159934	-10778	-
mir-153-1	TTCAGCACCGCGGACAGCGCCA	chr2:219998545-219998566	1060	PTPRN
mir-346	ATCAGTACCTCGGACAGCGCCA	chr10:88056588-88056609	59621	GRID1
mir-218-2	TTCAGAGCCCTGGCCATAGCCA	chr5:168520831-168520852	139703	SLIT3
mir-139	TTCAGCACCCTGGAGAGAGGCC	chr11:72065649-72065670	-2610	PDE2A
mir-95	TTCAGAACCAAGGCCACCTTGG	chr4:8205631-8205652	72958	ABLIM2
mir-455	CTCAGGACTCTGGACAGCTGTT	chr9:114005656-114005677	7873	COL27A1

**Table 2:** Enriched motifs in the regulatory regions of brain-related miRNAs

Motif	Conserved Num	Total number	Conservation rate	Neutral conservation rate	Z-score	Factor*	Factor consensus^†^	Similarity score^‡^
GACGTCA	20	33	0.61	0.069	11.7	CREB	TGACGTCA	0.95
CCATCTG	31	127	0.24	0.058	8.7	E47	AMCATCTGTT	0.93
ATAACCG	8	11	0.73	0.069	8.3			
AGACGCG	8	12	0.67	0.069	7.9			
TGAGTCA	20	83	0.24	0.058	6.9	Bach2	SRTGAGTCANC	0.97
AACAAAG	22	107	0.21	0.058	6.3	LEF-1	SWWCAAAGGG	0.81
AGATAAC	14	54	0.26	0.058	6.1	GATA-1	CWGATAACA	0.89
GCAGCTG	29	183	0.16	0.058	5.6	LBP-1	SCAGCTG	0.94
ATGCGCA	8	20	0.40	0.069	5.6			
CCTTTGT	17	82	0.21	0.058	5.6	LEF-1	CCCTTTGWWS	0.86
ACAGCAA	18	90	0.20	0.058	5.6			
ATGGCTT	17	84	0.20	0.058	5.5			
CTGCCAG	28	181	0.16	0.058	5.4			
GCGCCAT	7	17	0.41	0.069	5.4			
CGCACGC	7	17	0.41	0.069	5.4	AhR	CACGCNA	0.86
GGTGCTA	11	44	0.25	0.058	5.3			
CAATAAA	19	107	0.18	0.058	5.1			
GCGCGTC	8	23	0.35	0.069	5.1			
GTCTGTC	13	61	0.21	0.058	5.0	SMAD3	TGTCTGTCT	0.89
ATTAAGG	13	61	0.21	0.058	5.0	Nkx2-5	CAATTAWG	0.82
TGACAAG	13	63	0.21	0.058	4.9			
ATTAACT	12	56	0.21	0.058	4.9			
GGGATTA	10	42	0.24	0.058	4.8	PITX2	YTGGGATTANW	0.93
ATGCTAA	11	49	0.22	0.058	4.8	POU3F2	TTATGYTAAT	0.82
GCACAAA	13	64	0.20	0.058	4.8			
CCACCTG	22	144	0.15	0.058	4.7	MyoD	TNCNNCACCTG	0.88
AATTAAA	21	135	0.16	0.058	4.7	NKX6-1	AACCAATTAAAW	0.93
TGCAAAT	17	99	0.17	0.058	4.7	Oct1	TATGCAAAT	0.93
CTAATTG	8	31	0.26	0.058	4.6	S8	GNTAATTRR	0.86
CGCTGAC	7	21	0.33	0.069	4.6			
CACCAGG	18	110	0.16	0.058	4.6			
TCAATAA	13	68	0.19	0.058	4.6	HNF-6	HWAAATCAATAW	0.8
TTTGCAT	17	102	0.17	0.058	4.6	Oct1	ATTTGCATA	0.96

*Transcription factors from Transfac database. ^†^Known consensus in Transfac database that is similar to the 7-mer. ^‡^Measure the similarity between the 7-mer and the Transfac factor consensus. The score ranges from 0 to 1, with 1 for two identical consensus sequences.

**Table 3:** CRE sites present near a set of brain-related miRNAs in the human genome

	Conserved CRE site*		Conserved CRE half site^†^	
	
miRNA	Position^‡^	Distance (bp)	Position^‡^	Distance (bp)
mir-124a-1			chr8:9801040-9801044	-2648
mir-124a-2	chr8:65452347-65452354	-1913		
mir-124a-3	chr20:61279330-61279337	-968	chr20:61232305-61232309	-47992
			chr20:61276720-61276724	-3577
			chr20:61317969-61317973	37665
mir-9-1	chr1:153204718-153204725	-1423	chr1:153212345-153212349	-9051
mir-9-2	chr5:88007547-88007554	-9034	chr5:88016703-88016707	-18190
			chr5:87995510-87995514	3003
mir-9-3	chr15:87706692-87706699	-5565	chr15:87712302-87712306	50
	chr15:87711861-87711868	-391	chr15:87740065-87740069	27813
	chr15:87743860-87743867	31604	chr15:87757417-87757421	45165
			chr15:87757437-87757441	45185
mir-132/212	chr17:1901302-1901309	-1247	chr17:1922008-1922012	-21956
	chr17:1900538-1900545	-486	chr17:1921968-1921972	-21916
	chr17:1900522-1900529	-470	chr17:1913396-1913400	-13344
	chr17:1900084-1900091	-35		
mir-135a-2			chr12:96426695-96426699	-33363
mir-153-1	chr2:219999719-219999726	-15292	chr2:219969610-219969614	14817
	chr2:219939817-219939824	44611	chr2:219969479-219969483	14948
			chr2:219964362-219964366	20065
mir-29a/29b-1	chr7:130063683-130063690	-44859		
mir-29b-2			chr1:204385822-204385826	-21559
			chr1:204384854-204384858	-20591
mir-139			chr11:72021296-72021300	-17474

*CRE (cAMP response element); site: TGACGTCA. ^†^CRE half site: TGACG; can bind to CREB with weaker affinity. ^‡^Position is referenced on hg17. Only sites perfectly conserved in human, mouse, rat and dog are shown.
