# Supplementary material for: Comparative sequence analysis reveals an intricate network among REST, CREB and miRNA in mediating neuronal gene expression
Source: Genome Biol. 2006 Sep 26;7(9):R85. doi: 10.1186/gb-2006-7-9-r85 (PMC1794552; doi:10.1186/gb-2006-7-9-r85)
Supplement: Additional data file 1 — A PDF containing supporting figures and tables. [file gb-2006-7-9-r85-S1.pdf]

Supplementary Figures and Tables

Supplementary Figure 1 Distribution of the distance between the predicted NRSE sites and their the nearest genes. The distance is measured by the number of nucleotides located between a NRSE site and the transcriptional start site of its corresponding gene. Negative numbers indicate that NRSE sites are located upstream of the genes.

Supplementary Figure 2 A list of identified NRSE target genes that are specifically expressed in brain-related tissues. Each row represents the normalized expression levels of a gene across 61 mouse tissues. Please see the Methods section on how the expression values are normalized and on how the genes are selected.

Supplementary Figure 3 Gene expression of the predicted miRNA targets in 61 mouse tissues. (A), (B) and (C) show the expression of predicted targets for miR-124a, miR-132, and miR-9 respectively. Note that only a subset of genes that are specifically expressed in brain-related tissues is shown. The expression for all target genes is provided in the supplementary website. The expression values are normalized such that the mean expression values for each gene across different tissues is zero and standard deviation is 1. Please see the Methods section on the analysis of gene expression data and how the brain-related genes are selected.

Supplementary Figure 4 Conservation and EST evidences support a longer *REST* 3'UTR transcript. Shown are the annotations in this region from the UCSC genome browser. The predicted new 3'UTR is highlighted in yellow. Note that this region is packed with human ESTs, highly conserved even with chicken, and have few transposable elements.

Supplementary Figure 5 Expression of protein-coding genes hosting miRNAs in their introns. The expression for each gene is measured across 61 mouse tissues. The gene Slit3 is expressed at relative high level in amygdala, but with even much higher expression in fertilized egg. Slit3 is known to play a key role in the neural development, especially for the development of cranial motoneurons. It is possible that its high expression in fertilized egg might reflect its particular role implicated in neural development.

Supplementary Figure 6 Expression of 160 genes across different mouse tissues ordered by log-odds score of their corresponding NRSE sites.

Supplementary Table 1 A list of known NRSE sites and their conservation properties.

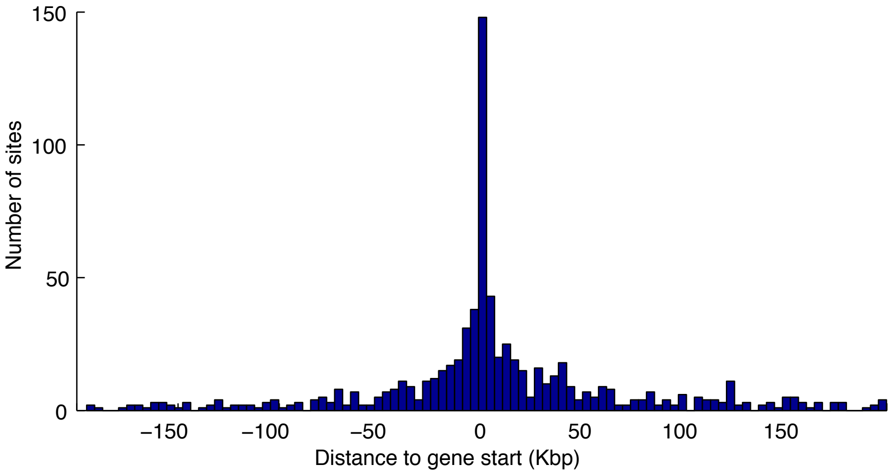

Supplementary Figure 1

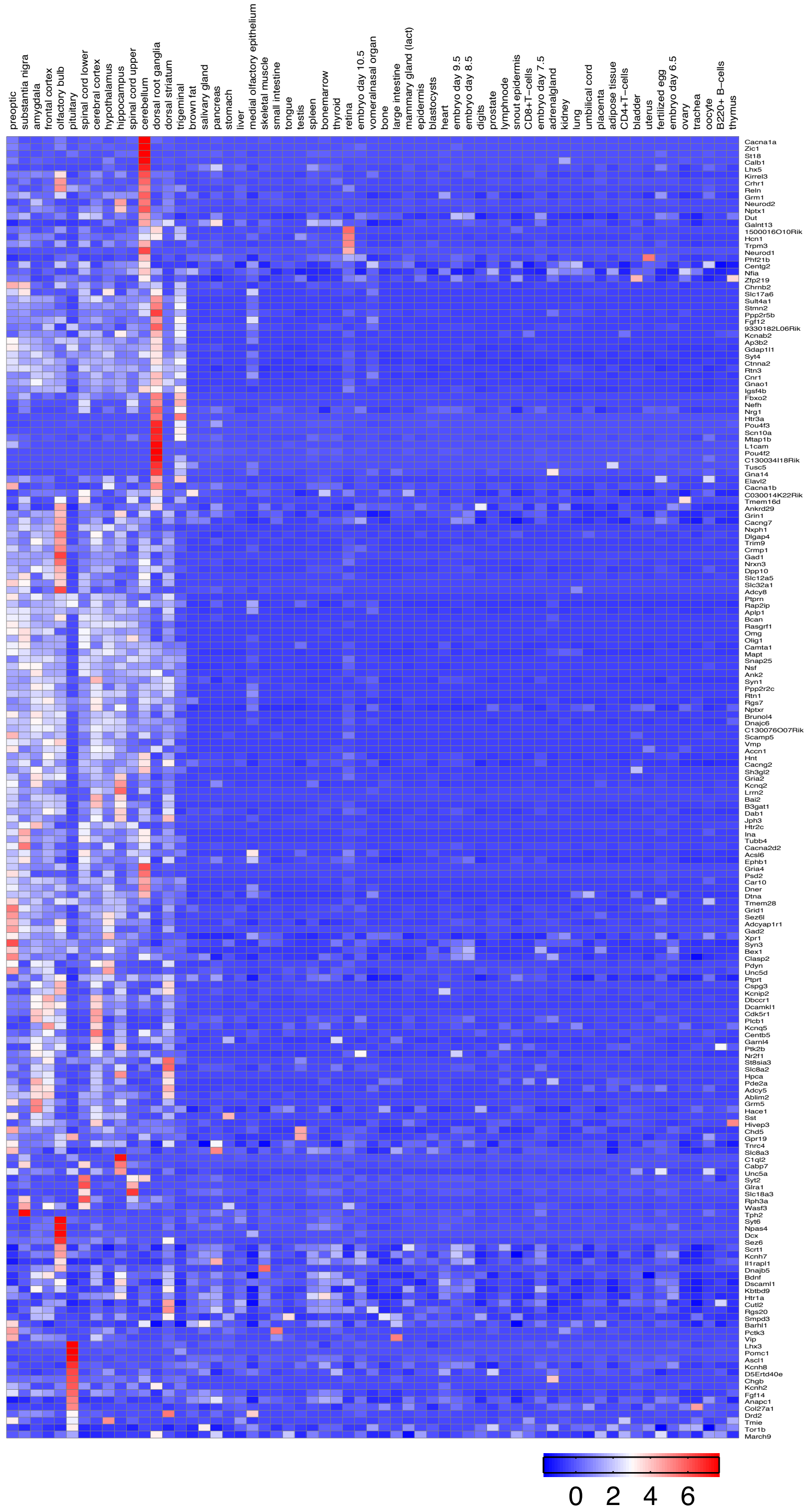

Supplementary Figure 2

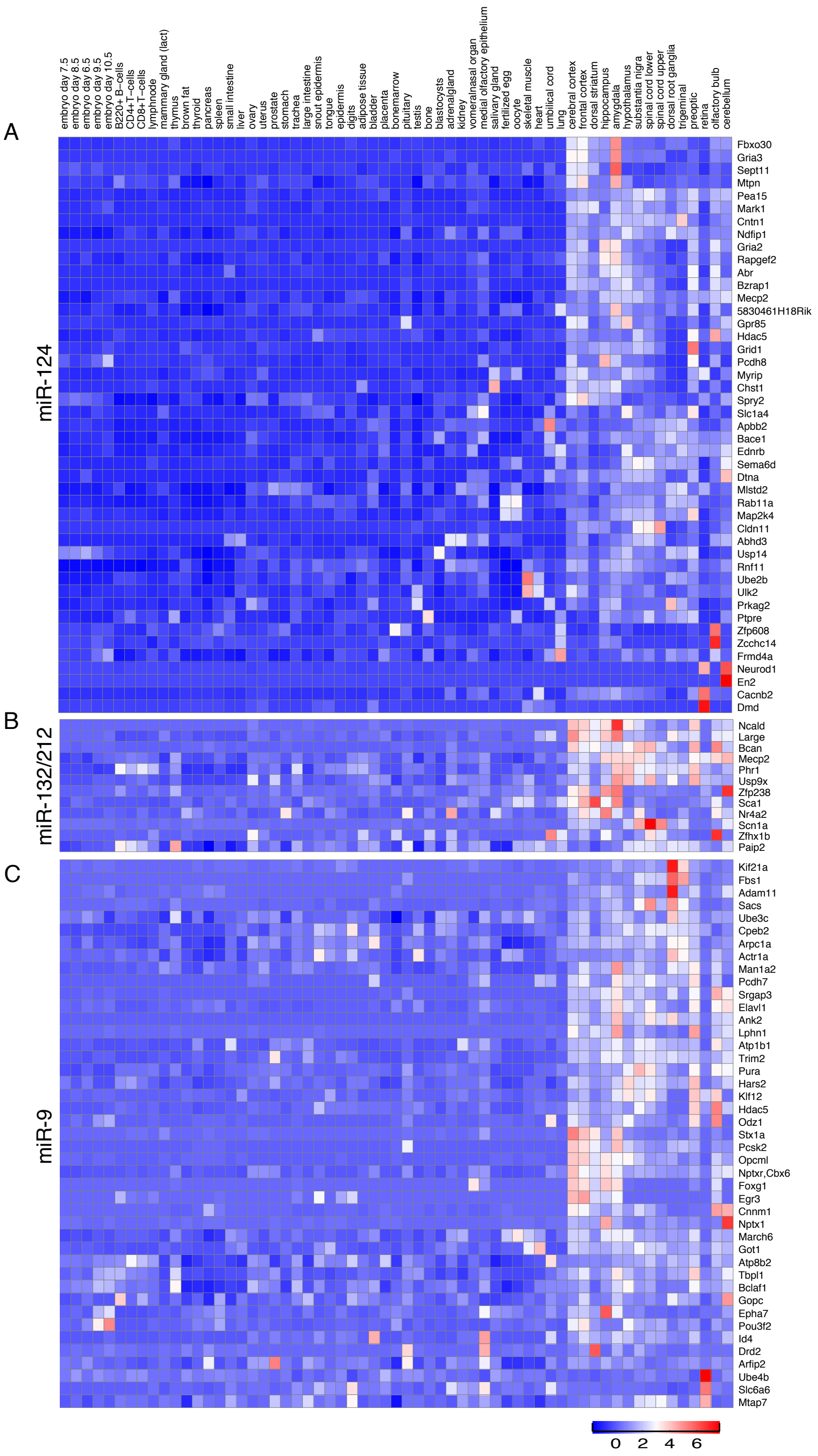

Supplementary Figure 3

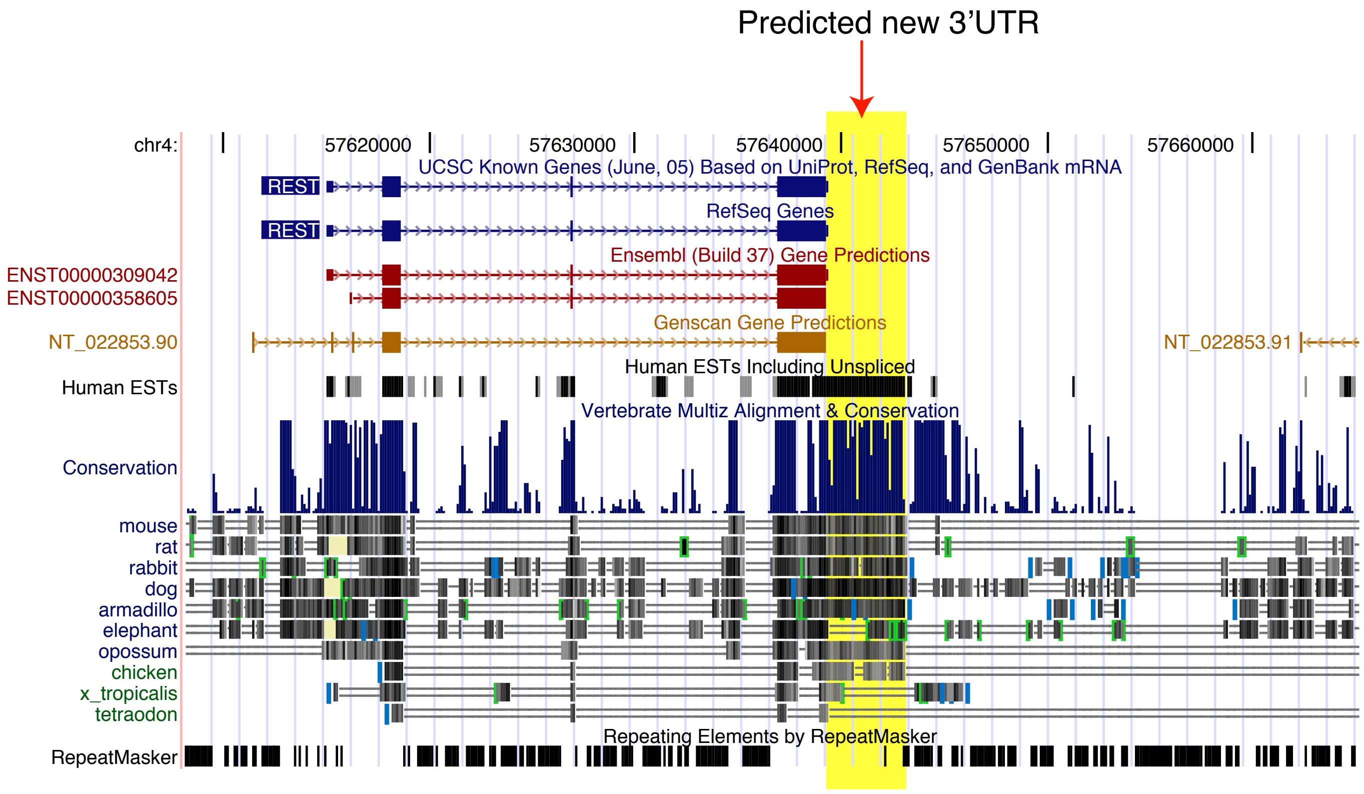

Supplementary Figure 4

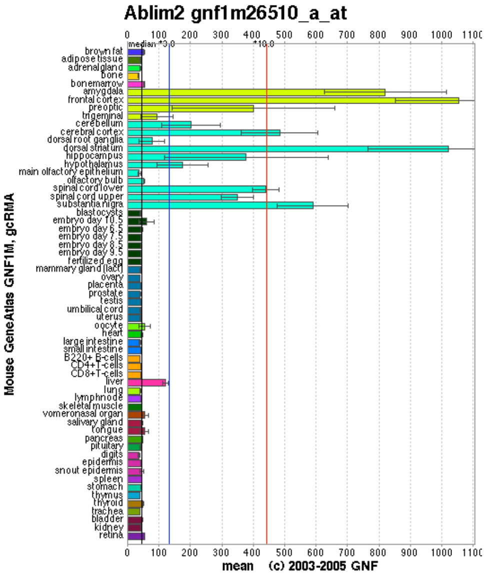

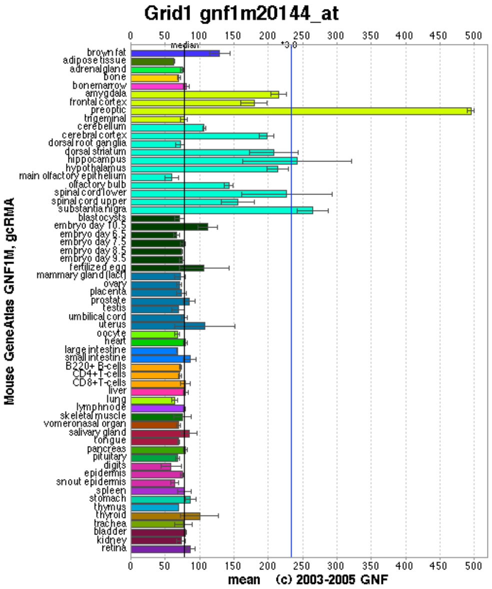

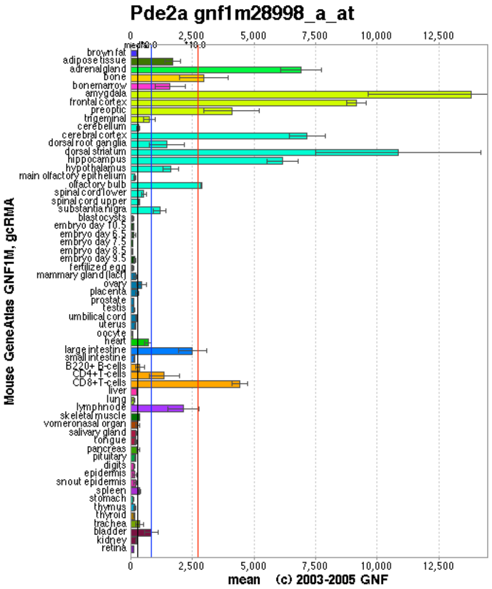

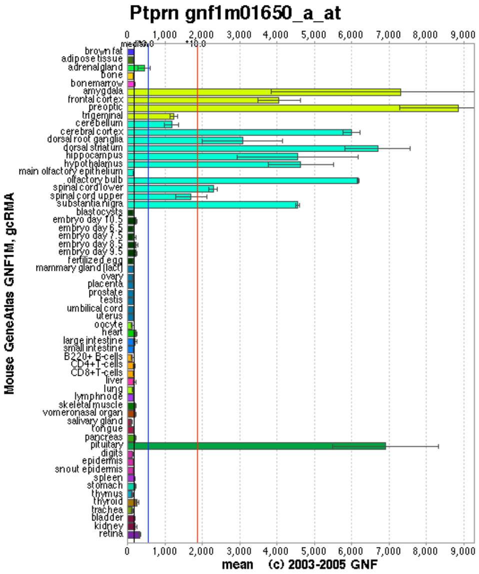

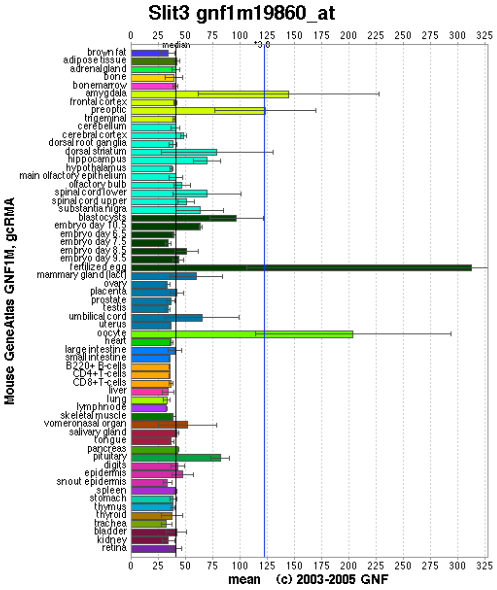

Supplementary Figure 5

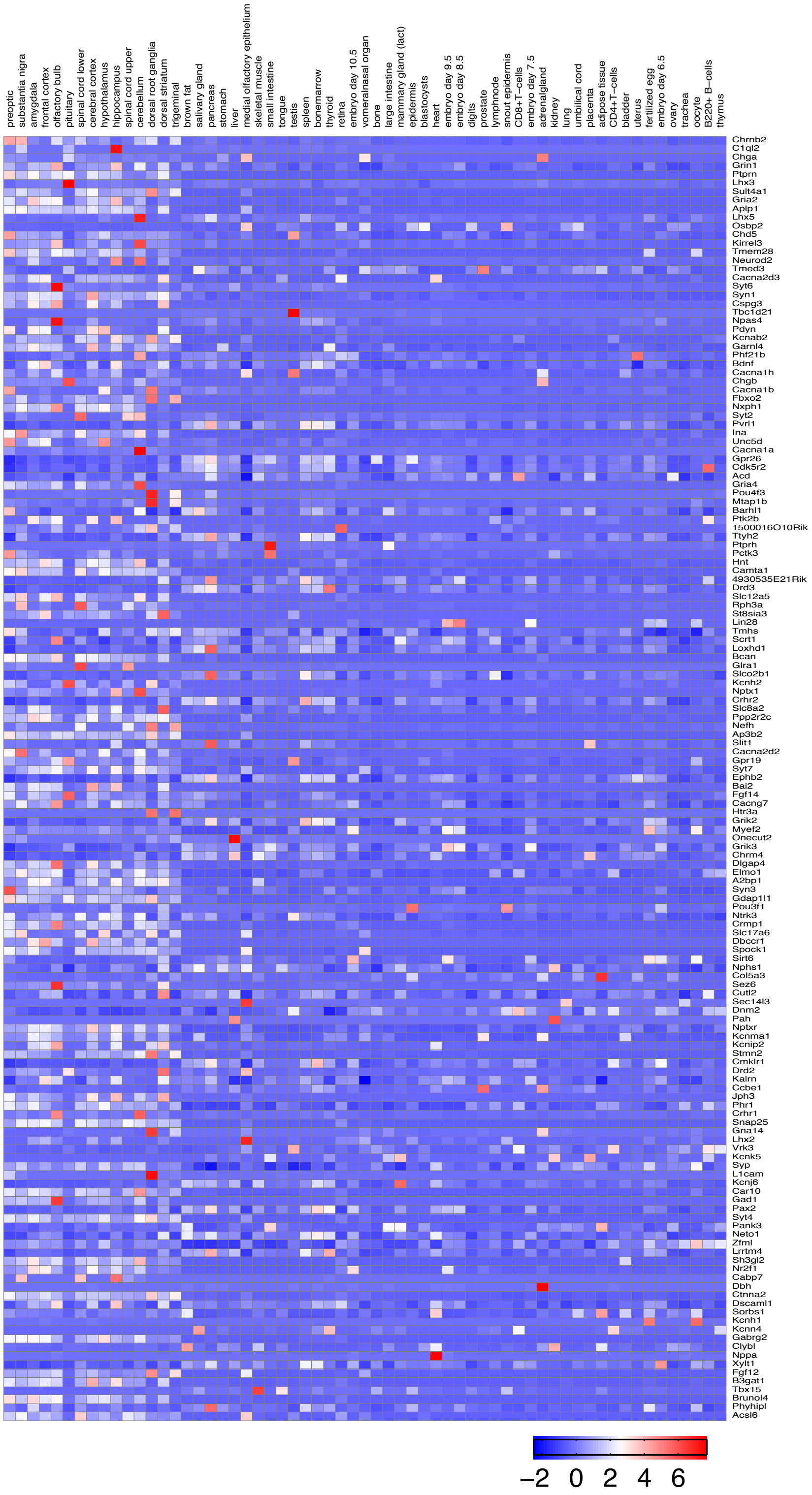

Supplementary Figure 6

Supplementary Table 1 A list of known RE1/NRSE sites and their conservation properties in human/mouse/rat/dog

Sequence	Mutation in the first 17 bases	Mutation in posititions 1 to 8 and 11 to 16	Difference from NRSE consensus	Log-odds score	Genome coordinates and strand
>nrse_chr11_27698819_m BDNF					
TTCAGCACCTTGGACAGAGCC	0	0	0	31.4	hg17.chr11:27698799-27698819,-
TTCAGCACCTTGGACAGAGCC	0	0	0	31.6	mm5.chr2:109567568-109567588,+
TTCAGCACCTTGGACAGAGCC	0	0	0	31.6	rn3.chr3_random:930771-930791,+
TTCAGCACCTTGGACAGAGCC	0	0	0	31.4	canFam1.chr21:50514521-50514541,-
>nrse_chr8_80684341_p STMN2					
TTCAGCACCAAGGAGAGTGCC	0	0	0	27.9	hg17.chr8:80684341-80684361,+
TTCAGCACCACGGACAGCGCT	2	1	0	33.8	mm5.chr3:8544331-8544351,+
TTCAGCACCACGGAGAGTGCC	1	0	0	30.0	rn3.chr2:95279221-95279241,-
TTCAGCACCAAGGAGAGTGCC	0	0	0	27.9	canFam1.chr29:30067518-30067538,+
>nrse_chr2_165920501_p SCN2A2					
CTCAGCACCATGGACAGCGTT	0	0	0	27.4	hg17.chr2:165920501-165920521,+
TTCAGCACCACGGACAGCACC	2	0	0	34.0	mm5.chr2:65558600-65558620,+
TTCAGAACCACGGACAGCACC	3	1	0	30.5	rn3.chr3:47483668-47483688,+
TTCAGCACCACGGACAGCGTT	2	0	0	31.1	canFam1.chr36:13584742-13584762,+
>nrse_chrX_152661866_m L1CAM					
GTCAGGACCATGGACAGGGAC	0	0	1	23.9	hg17.chrX:152661846-152661866,-
GTCAGCACCATGGACAGGAAC	1	1	0	26.6	mm5.chrX:65427921-65427941,-
GTCAGCACCATGGACAGGAAC	1	1	0	26.6	rn3.chrX:159876613-159876633,-
gTCAGCACCACGGACAGGGGA	2	1	0	25.9	canFam1.chrX:124778187-124778207,-
>nrse_chrX_152661824_m L1CAM					
TTCAGCACCAGGGACAGCGAA	0	0	0	27.6	hg17.chrX:152661804-152661824,-
TCCAGCACCACGGACAGCAGA	2	1	1	25.0	mm5.chrX:65427891-65427911,-
TCCAGTACCACGGACAGCAGA	3	2	2	19.8	rn3.chrX:159876580-159876600,-
TTCAGGACCACGGACAGCGGG	2	1	1	26.1	canFam1.chrX:124778158-124778178,-
>nrse_chr20_10148269_p SNAP25					
CTCAGCACCCTGGATAGCAAC	0	0	1	21.1	hg17.chr20:10148269-10148289,+
CTCAGCACCCTGGACAGGAGT	1	1	0	23.0	mm5.chr2:136463311-136463331,+
CTCAGCACCCTGGACAGGAGT	1	1	0	23.0	rn3.chr3:124712985-124713005,+
GTCAGCACCCTGGACAGCGAT	2	1	0	25.8	canFam1.chr24:15095171-15095191,-
>nrse_chr20_10148320_p SNAP25					
TTCAGCACCGGGGACAGCGGT	0	0	0	27.7	hg17.chr20:10148320-10148340,+
TTCAGCACCGCGGACAGCGAT	1	0	0	31.3	mm5.chr2:136463361-136463381,+
TTCAGCACCGCGGACAGCGAT	1	0	0	31.3	rn3.chr3:124713035-124713055,+
TTCAGCACCGCGGAGAGCGGC	2	1	0	29.7	canFam1.chr24:15095120-15095140,-
>nrse_chr9_137309549_p GRIN1					
TTCAGCACCGCGGACAGCGCC	0	0	0	34.5	hg17.chr9:137309549-137309569,+
TTCAGCACCTCGGACAGCACC	1	0	0	32.4	mm5.chr2:25279036-25279056,-
TTCAGCACCTCGGACAGCATC	1	0	0	30.0	rn3.chr3:3480288-3480308,-
TTCAGCACCGCGGACAGCGCC	0	0	0	34.5	canFam1.chr9:41044966-41044986,-
>nrse_chr5_151284508_p GLRA1					
TTCAGCACCACGGAGAGCGTC	0	0	0	29.9	hg17.chr5:151284508-151284528,+
TTCAGCACCACGGAGAGCGTC	0	0	0	30.2	mm5.chr11:55247677-55247697,+
TTCAGCACCACGGAGAGCGTC	0	0	0	30.2	rn3.chr10:41035413-41035433,+
TTCAGCACCACGGAGAGCGTC	0	0	0	29.9	canFam1.chr4:60614365-60614385,-
>nrse_chr1_151353503_p CHRNB2					
TTCAGCACCACGGACAgcgcc	0	0	0	35.4	hg17.chr1:151353503-151353523,+
TTCAGCACCACGGACAGCGCT	0	0	0	33.8	mm5.chr3:90189446-90189466,-
TTCAGCACCACGGACAGCGCT	0	0	0	33.8	rn3.chr2:181919553-181919573,-
TTCAGCACCGCGGACAGCGCC	1	0	0	34.5	canFam1.chr7:45410505-45410525,-
>nrse_chr8_91164252_m CALB1					
AGCAGCACCGAGGACAGCGCC	0	0	1	25.2	hg17.chr8:91164232-91164252,-
AGCAGCACCGCGGACAGCGCC	1	0	1	27.6	mm5.chr4:15808391-15808411,+
AGCAGCACCGCGGACAGCGCC	1	0	1	27.6	rn3.chr5:30455674-30455694,+
CTCAGCACCGAGGACAGCTCC	2	1	0	28.0	canFam1.chr29:38572206-38572226,-
>nrse_chr1_11840056_m NPPA					
TTCAGCACCATGGACAGAAGA	0	0	0	26.6	hg17.chr1:11840036-11840056,-
TTCAGCACCAAGGACAGACGC	1	0	0	26.7	mm5.chr4:145887995-145888015,+
TTCAGCACCACGGACAGACGC	1	0	0	28.6	rn3.chr5:165085986-165086006,+
TTCAGCACCACGGACAGCAGC	1	0	0	32.1	canFam1.chr2:86623257-86623277,+
>nrse_chr1_6046932_p KCNAB2					
TTCAGCACCTCGGACAGCGCT	0	0	0	31.8	hg17.chr1:6046932-6046952,+
TTCAGCACCCAGGACAGCTCC	2	0	0	30.2	mm5.chr4:150310481-150310501,-
TTCAGCACCCAGGACAGCTCC	2	0	0	30.2	rn3.chr5:169668695-169668715,-

TTCAGCATCTGGGGCAGCACT	3	2	2	16.4	canFam1.chr5:63032964-63032984,+
>nrse_chr2_219652224_m CDK5R2					
CTCAGCACCTCGGACAGTTCC	0	0	0	26.5	hg17.chr2:219652204-219652224,-
CTCAGCACCTCGGACAATTCC	1	1	1	21.8	mm5.chr1:75317485-75317505,-
CTCAGCACCTCGGACAATTCC	1	1	1	21.8	rn3.chr9:74340946-74340966,-
ATCAGCACCTCAGACAGCTCC	2	1	0	23.5	canFam1.chr37:28510580-28510600,-
>nrse_chr2_219653264_m CDK5R2					
TTCAGCACCACGGACAGCGAA	0	0	0	30.8	hg17.chr2:219653244-219653264,-
TTCAGCACCACGGACAGCGAA	0	0	0	31.1	mm5.chr1:75318462-75318482,-
TTCAGCACCACGGACAGCGAA	0	0	0	31.1	rn3.chr9:74341934-74341954,-
ATCAGCACCACGGACAGCGAA	1	0	0	28.0	canFam1.chr37:28511567-28511587,-
>nrse_chr19_19186936_p CSPG3					
TTCAGCACCACGGACAGGTCC	0	0	0	32.0	hg17.chr19:19186936-19186956,+
TTCAGCACCAAGGACAGATCG	1	0	0	26.8	mm5.chr8:69152990-69153010,-
TTCAGCACCAAGGACAGGTCC	1	0	0	30.5	rn3.chr16:19788177-19788197,+
TTCAGCACCACGGATAGGTCC	1	1	1	26.8	canFam1.chr20:46827133-46827153,-
>nrse_chr1_111001541_m KCNA3					
TTCAGCACCAAGGACAGAGAC	0	0	0	30.4	hg17.chr1:111001521-111001541,-
TTCAGCACCATGGACAAGGGT	2	1	1	24.5	mm5.chr3:107342354-107342374,+
TTCAGCACCAAGGACAGGGTG	0	0	0	26.4	rn3.chr2:202353732-202353752,+
TTCAGCACCAAGGACAGAAGC	0	0	0	28.6	canFam1.chr6:42825633-42825653,+
>nrse_chr3_115380519_m DRD3					
TTCAGCACCAAGGACAGAACC	0	0	0	30.2	hg17.chr3:115380499-115380519,-
CTCAGCACCAAGGACAGAGAG	1	0	0	24.1	mm5.chr16:43630422-43630442,+
CTCAGCACCAAGGACAGAGAG	1	0	0	24.1	rn3.chr11:58587125-58587145,-
TTCAGCACCAAGGACAGTGCC	0	0	0	31.2	canFam1.chr33:21483747-21483767,-
>nrse_chr9_136322946_m LHX3					
TTCAGCACCGCGGACAGCGCC	0	0	0	34.5	hg17.chr9:136322926-136322946,-
TTCAGCACCGCGGACAGCGCC	0	0	0	35.1	mm5.chr2:26168294-26168314,-
TTCAGCACCGCGGACAGCGCC	0	0	0	35.1	rn3.chr3:4389191-4389211,-
TTCAGCACCGCGGACAGCGCC	0	0	0	34.5	canFam1.chr9:41786607-41786627,+
>nrse_chr12_112380809_m LHX5					
TTCAGCACCGCGGACAGCTCC	0	0	0	32.7	hg17.chr12:112380789-112380809,-
TTCAGCACCGCGGACAGCTCC	0	0	0	33.2	mm5.chr5:117915809-117915829,+
TTCAGCACCGCGGACAGCTCC	0	0	0	33.2	rn3.chr12:37203815-37203835,-
ATCAGCACCGCGGACAGCTCC	1	0	0	29.9	canFam1.chr26:13956517-13956537,-
>nrse_chr13_101369969_p FGF14					
TTCAGCACCGCGGACAGGGAT	0	0	0	29.2	hg17.chr13:101369969-101369989,+
TTCAGCACCGCGGACAGTGAC	0	0	0	30.8	mm5.chr14:116491551-116491571,+
TTCAGCACCGTGGACAGTGAC	1	0	0	29.9	rn3.chr15:109316548-109316568,+
TTCAGGACCGAGGACAGGGAT	2	1	1	23.3	canFam1.chr22:54512919-54512939,+
>nrse_chr14_92459621_p CHGA					
TTCAGCACCGCGGACAGCGCC	0	0	0	34.5	hg17.chr14:92459621-92459641,+
TTCAGCACCTCGGACAGCACC	1	0	0	32.4	mm5.chr12:97233700-97233720,+
TTCAGCACCTCGGACAGCACC	1	0	0	32.4	rn3.chr6:126825403-126825423,+
TTCAGCACCGCGGACAGCGCC	0	0	0	34.5	canFam1.chr8:4944156-4944176,+
>nrse_chr2_219998566_m PTPRN					
TTCAGCACCGCGGACAGCGCC	0	0	0	34.5	hg17.chr2:219998546-219998566,-
TTCAGAACCATGGACAGCGCC	3	1	0	31.5	mm5.chr1:75709818-75709838,-
TCCAGCACCACGGACAGCATC	2	1	1	27.3	rn3.chr9:74688760-74688780,-
TTCAGCACCGCGGACAGCGCC	0	0	0	34.5	canFam1.chr37:28832304-28832324,-
>nrse_chr5_161428260_p GABRG2					
TTCAGCACCAGGGACAGCAGT	0	0	0	26.9	hg17.chr5:161428260-161428280,+
TTCAGCACCAAGGACAGCGGT	1	0	0	30.3	mm5.chr11:41639386-41639406,-
TTCAGCACCAAGGACAGCGAT	1	0	0	30.2	rn3.chr10:27126446-27126466,-
TTCAGCACCAGGGACAGCAGT	0	0	0	26.9	canFam1.chr4:52070373-52070393,-
>nrse_chr1_11853814_m NPPB					
ATCAGCACCACGGACAGCGGC	0	0	0	31.0	hg17.chr1:11853794-11853814,-
ATCAGAACCACGGACAGCTAC	1	1	0	25.9	mm5.chr4:145871317-145871337,+
ATCAGAACCATGGACAGCTAC	2	1	0	25.1	rn3.chr5:165071977-165071997,+
CTCAGCACCACGGACAGCGGC	1	0	0	30.8	canFam1.chr2:86610700-86610720,+
>nrse_chr1_207301544_p KCNH1					
ATCAGCACCCAGGACAGCATC	0	0	0	24.8	hg17.chr1:207301544-207301564,+
GTCAGCACCCTGGACAGCTCC	2	0	0	28.1	mm5.chr1:192353285-192353305,-
GTCAGTACCCTGGACAGCATC	3	1	1	20.5	rn3.chr13:108549570-108549590,-
ATCAGCACCCTGGACAGAGTG	1	0	0	22.3	canFam1.chr7:11912396-11912416,+
>nrse_chr1_207288522_p KCNH1					
TTCAGAACCATGGACAGTTCC	0	0	0	27.0	hg17.chr1:207288522-207288542,+
TTCAGGACCATGGACAGTTCT	1	1	1	24.2	mm5.chr1:192367627-192367647,-
TTCAGGACCACGGACAGTGCC	2	1	1	29.0	rn3.chr13:108557186-108557206,-

TTCAGCACCCTGGACAGTGCC	2	1	0	30.1	canFam1.chr7:11900079-11900099,+
>nrse_chr15_68055979_m TLE3					
TTCAGAACCATGGACAGAGGC	0	0	0	27.9	hg17.chr15:68055959-68055979,-
TTCAGAACCATGGACAGAGGC	0	0	0	28.1	mm5.chr9:61707118-61707138,+
TTCAGAACCATGGACAGAGGC	0	0	0	28.1	rn3.chr8:65617619-65617639,+
TTCAGAACCATGGACAGAGGC	0	0	0	27.9	canFam1.chr30:36732582-36732602,-
>nrse_chr22_35364989_m CACNG2					
TTCAGCACCTTGGAGAGCATC	0	0	0	25.8	hg17.chr22:35364969-35364989,-
TTCAGCACCTTGGAGAGCTCC	0	0	0	28.3	mm5.chr15:78393924-78393944,-
TTCAGCACCTTGGAGAGCACC	0	0	0	28.3	rn3.chr7:116018162-116018182,-
TTCAGCACCAAGGAGAGCACC	2	0	0	28.7	canFam1.chr10:30977690-30977710,+
>nrse_chr10_120345766_p GPR10					
GTCAGCACCACGGACAGCTTC	0	0	0	28.1	hg17.chr10:120345766-120345786,+
ATCAGCACCACGGGCAGCTCC	2	1	1	26.6	mm5.chr19:59860036-59860056,+
ATCAGCACCTCGGGCAGCTCC	3	1	1	25.0	rn3.chr1:267281791-267281811,+
TTCAGCACAGCGGACAGCTCC	3	1	1	27.0	canFam1.chr28:31995439-31995459,+
>nrse_chr13_77035503_m SCEL					
TTCAGCACCGTGGACAGGGTT	0	0	0	27.8	hg17.chr13:77035483-77035503,-
TTCAGCACCATGGACAGGCAC	1	0	0	27.7	mm5.chr14:95781930-95781950,-
TTCAGCACCATGGACAGGCAC	1	0	0	27.7	rn3.chr15:87589807-87589827,-
TTCAGCACCGTGGACAGGGTT	0	0	0	27.8	canFam1.chr22:34020238-34020258,-
>nrse_chr22_37545209_m NPTXR					
TTCAGCACCCTGGACAGGGCA	0	0	0	27.9	hg17.chr22:37545189-37545209,-
TTCAGCACCCTGGACAGAGCA	0	0	0	28.2	mm5.chr15:80119289-80119309,-
TTCAGCACCACGGACAGGGCG	2	0	0	30.7	rn3.chr7:118063259-118063279,-
TTCAGCACCCTGGACAGGGCA	0	0	0	27.9	canFam1.chr10:29181384-29181404,+
>nrse_chr7_150118828_m KCNH2					
TTCAGCACCTTGGACAGAGAC	0	0	0	29.8	hg17.chr7:150118808-150118828,-
TTCAGCACCTCGGACAGAGGC	1	0	0	30.9	mm5.chr5:22755639-22755659,-
TTCAGCACCTTGGACAGAGAC	0	0	0	29.9	rn3.chr4:6186606-6186626,+
TTCAGCACCTTGGACAGAGAC	0	0	0	29.8	canFam1.chr16:16883946-16883966,-
>nrse_chr20_5840350_p CHGB					
GTCAGCACCGCGGACAGCGCC	0	0	0	31.2	hg17.chr20:5840350-5840370,+
TTCAGCACCCCGGACAGCTCC	2	0	0	32.1	mm5.chr2:132530817-132530837,+
TTCAGAACCTCGGACAGCTCC	3	1	0	28.9	rn3.chr3:120477432-120477452,+
ATCAGCACCGCGGACAGCGCT	1	0	0	29.7	canFam1.chr24:18944570-18944590,-
>nrse_chr22_28199238_m NEFH					
TTCAGCACCGGGGACAGGGCC	0	0	0	29.6	hg17.chr22:28199218-28199238,-
TTCAGCACCACGGACAGCAGC	2	0	0	32.4	mm5.chr11:4844186-4844206,+
TTCAGAACCATGGACAGCGCC	3	1	0	31.5	rn3.chr14:85625288-85625308,+
TTCAGCACCAAGGACAGGGCC	2	0	0	32.0	canFam1.chr26:25862535-25862555,-
>nrse_chr15_86510797_m NTRK3					
TTCAGCACCATGGCCAGAGCC	0	0	0	28.5	hg17.chr15:86510777-86510797,-
TTCAGCACCATGGCCAGGGCT	0	0	0	26.9	mm5.chr7:65560136-65560156,-
TTCAGCACCATGGCCAGGGCT	0	0	0	26.9	rn3.chr1:134296513-134296533,-
TTCAGCACCATGGCCAGAGCC	0	0	0	28.5	canFam1.chr3:54375781-54375801,-
>nrse_chr11_61091418_m SYT7					
TTCAGCACCTTGGACAGCTCT	0	0	0	29.3	hg17.chr11:61091398-61091418,-
TTCAGTACCAAGGGCAGCACC	4	2	2	22.3	mm5.chr19:9502400-9502420,+
TTCAGTACCACGGGCAGCACC	4	2	2	24.2	rn3.chr1:213048931-213048951,+
TTCAGCACCACGGACAGATCC	2	0	0	32.0	canFam1.chr18:65203709-65203729,+

Note:
*Known NRSE consenusus: NTYAGMRCCNNRGMSAG
*Mutation: the number of nucleotides different from the human sequence
*Aligned sequences are in the order of human, mouse, rat and dog
